# Experiences with and attitudes towards geriatric screening among older emergency department patients: a qualitative study

**DOI:** 10.1186/s12877-021-02144-7

**Published:** 2021-03-20

**Authors:** Laura C. Blomaard, Mareline Olthof, Yvette Meuleman, Bas de Groot, Jacobijn Gussekloo, Simon P. Mooijaart

**Affiliations:** 1 Department of Internal Medicine, section Geriatrics, Leiden University Medical Center, PO Box 9600, 2300 RC, Leiden, The Netherlands; 2 Department of Public Health and Primary Care, Leiden University Medical Center, Leiden, The Netherlands; 3 Department of Clinical Epidemiology, Leiden University Medical Center, Leiden, The Netherlands; 4 Department of Emergency Medicine, Leiden University Medical Center, Leiden, The Netherlands; 5 Institute of Evidence-Based Medicine in Old Age | IEMO, Leiden, The Netherlands

**Keywords:** Frailty, Frailty screening, Emergency medicine, Older adults, Qualitative research

## Abstract

**Background:**

The patient perspective on the use of screening for high risks of adverse health outcomes in Emergency Department (ED) care is underexposed, although it is an important perspective influencing implementation in routine care. This study explores the experiences with, and attitudes towards geriatric screening in routine ED care among older people who visited the ED.

**Methods:**

This was a qualitative study using individual face-to-face semi-structured interviews. Interviews were conducted in older patients (≥70 years) who completed the ‘Acutely Presenting Older Patient’ screener while visiting the ED of a Dutch academic hospital. Purposive convenience sampling was used to select a heterogeneous sample of participants regarding age, disease severity and the result from screening. Transcripts were analyzed inductively using thematic analysis.

**Results:**

After 13 interviews (7 women, median age 82 years), data saturation was reached. The participants had noticed little of the screening administration during triage and screening was considered as a normal part of ED care. Most participants believed that geriatric screening contributes to assessing older patients holistically, recognizing geriatric problems early and comforting patients with communication and attention. None of the participants had a negative attitude towards screening or thought that screening is discrimination on age. Care providers should communicate respectfully with frail older patients and involve them in decision-making.

**Conclusions:**

Older patients experienced geriatric screening as a normal part of ED care and had predominantly positive attitudes towards its use in the ED. This qualitative study advocates for continuing the implementation of geriatric screening in routine ED practice.

**Supplementary Information:**

The online version contains supplementary material available at 10.1186/s12877-021-02144-7.

## Introduction

Screening for high risks of adverse health outcomes in the Emergency Department (ED) has been advocated by various healthcare organizations, but the experiences and attitudes regarding geriatric screening among older people who visit the ED are unknown. In the last years, identification of frailty in the ED has received more attention and the use of screening tools is strongly promoted to enhance awareness and understanding of geriatric patients beyond their ED presenting complaint [1–3]. Geriatric screening in routine ED care, however, remains scarce and there is still an ongoing public debate on the pros and cons of screening in general [4, 5]. Geriatric screening is intended to assist in clinical decision making and to protect older people against age-based rationing of care [6, 7]. However, it is also feared that the label ‘frail’ can lead to unintended ageism [8]. So far, little attention has been paid to the perspectives of older people themselves and how they experience undergoing geriatric screening in general [9, 10], and experiences of older people with geriatric screening in the ED setting has not been studied before.

The consumer’s perspective – in this case, of older ED patients – is often underexposed, and because it can be different from the provider or organizational perspective, it is an important perspective influencing the implementation and effects of programs [11]. Therefore, there is a need for qualitative research exploring the older people’s perspective on geriatric screening in the ED. [2, 8] First, because if older people have a positive attitude towards geriatric screening in the ED and they believe it has added value, this might advocate for continuing the implementation of screening in routine practice. And second, because the experiences of older people could be used to further improve geriatric screening (administration) and better the field of geriatric emergency medicine in general.

Therefore, the aim of the present study was to explore the experiences with, and attitudes towards geriatric screening in routine ED care among older people who visited the ED using qualitative research methods.

## Methods

### Study design and participants

Within this explorative qualitative study, individual face-to-face semi-structured interviews were conducted between September 2019 and January 2020 in the Netherlands. The target study population was comprised of older people aged 70 years or older who had recently visited the ED of the Leiden University Medical Center (LUMC) and had completed the Acutely Presenting Older Patient (APOP) screener during their stay in the ED. Patients without treatment in the ED, patients who were not screened with the APOP screener, or patients who deceased before inclusion were excluded. Purposive sampling was applied to ensure a heterogeneous sample of patients with regard to gender, disease severity and APOP screening result. Patients who lived close to the research location were invited for participation for convenience purposes. Interviews were conducted until data saturation was reached and no additional information or themes were observed in the data. It was expected that data saturation would be reached after around 10–15 interviews [12, 13].

### The APOP screening program

The APOP screening program is developed for ED patients aged ≥70 years, and consists of a screening instrument and tailored interventions (detailed descriptions in Additional file 1) [14, 15]. This program has been implemented in routine ED care in the LUMC since March 2018 and triage nurses are instructed to screen all older patients during routine triage [16, 17]. The experiences of triage nurses who execute the screening in the ED has been described previously [16]. The APOP screening instrument consists of 9 questions (i.e. about physical functioning and cognition) and can be administered within 2 min. The instrument identifies patients at risk of 90-day functional decline and/or mortality and signs of impaired cognition [18]. A universally accepted definition of frailty does not exists, but frailty is most often defined as an aging-related syndrome of physiological decline, characterized by marked vulnerability to adverse health outcomes [19, 20]. We did not share a definition with the participants, because we were interested in their personal definition and perception of frailty. Since frailty is known to be associated with high risks of adverse health outcomes, we used the APOP risk stratification instrument which identifies older patients at high risk of adverse outcomes as a proxy for frailty. Patients were considered as ‘frail’ when having a 45% or higher risk of functional decline and/or mortality (‘high risk on functional domain’) or when having signs of impaired cognition (‘high risk on cognitive domain’). For patients with a ‘high risk’ screening result, interventions to increase comfort, family involvement and delirium prevention are executed in the ED. A complete comprehensive geriatric assessment is executed in patients who are hospitalized. Patients receive a telephone call within 24 h after discharge and the general practitioner is informed about the screening result.

### Procedures

Two female researchers, LCB (MD, PhD candidate) and MO (MSc Vitality and Ageing, PhD candidate), conducted the interviews, transcribed the recordings and performed the analyses. There was no treatment relationship between the researchers and the participants prior or after the study.

The interviews were planned within 1 month after patients’ ED visit. We chose this period as a trade-off between sufficient recovery time and minimalized recall bias. Eligible participants received an invitation letter by mail within 3 days after their ED visit or within 3 days after discharge from the hospital if they had been hospitalized. One week after sending the invitation letter, participants were invited by telephone to participate by one of the researchers. The appointment for the interview was preferably made within 2 weeks after the telephone call. Participants received a confirmation letter of the appointment with additional information regarding the study procedure, anonymity, and confidentiality. All participants were aware of the goals and reasons of the researchers for doing this study, and agreed to recording and anonymous usage of the data. All participants gave written informed consent before taking part in the interview. People who were not able to consent themselves (i.e. due to cognitive disorders) were not included. This study was performed in accordance with the Declaration of Helsinki and was approved by the Medical Ethical committee of the LUMC (Protocol nr. P17.165).

Based on existing literature and the formulated study objective, an interview guide was created to maintain consistency in the format of the interviews (see Additional file 2). This interview guide consisted of four themes (1. Experiences of the ED visit, 2. Experiences with geriatric screening in the ED, 3. Attitude towards geriatric screening in the ED, and 4. Needs and goals of older patients in the ED), followed by open-ended questions. Responses were further explored using additional questions and probes. After exploring the experiences of participants with screening, an informative video about the content of the APOP screening program was shown in order to help participants to generate an opinion about the use of such a program [21]. Because we expected that some participants might not be able to distinguish the screening questions from routine triage, the video was used to provide all participants with the same level of knowledge about the content of the screening program before exploring their attitude towards it. Participants were asked about their definition of frailty and the perception of their own frailty since we hypothesized that this might influence their attitude towards screening. One pilot interview was performed by the two main researchers to evaluate the interview guide for completeness and, if necessary, to make adjustments. All subsequent interviews were performed by one researcher individually. After every three interviews, the researchers discussed their findings and additional participants were recruited.

The interviews were conducted at the participants’ homes and lasted between 45 and 60 min. Field notes were made during the interviews. Although family members were not actively recruited for participation, they were welcome to attend and participate in the interview. Quotes of family members were occasionally used to add context to the statements of the patients.

### Analysis

All interviews were audio recorded and transcribed verbatim by the two main researchers. Data was anonymized and confidentiality was ensured by using codes instead of personal names in the transcriptions. Transcripts were analyzed inductively using thematic analysis. All transcripts were coded by both researchers and discussed to align coding strategy and judge consistency of interpretation. After open coding, the researchers used axial coding and developed a coding tree without the use of a pre-existing coding frame, by constant comparison, grouping similar themes and organizing them hierarchically. To ensure triangulation, the two main researchers discussed the preliminary themes with four other researchers (YM (PhD, medical psychologist), BdG (MD, PhD, emergency physician), JG (MD, PhD, professor primary care) and SPM (MD, PhD, internist geriatrician)). Finally, conceptual links and patterns among themes were derived from the data. All audio recordings, field notes and coded data were saved on a secured server and an audit trail was kept during the study project. The transcriptions were coded using Atlas.ti software version 8. The Consolidated Criteria for Reporting Qualitative Studies (COREQ) checklist was used to report the study (Additional file 3).

To describe patients’ characteristics, descriptive statistics were computed using data obtained from the hospital electronic health records. Data are presented as medians with ranges or numbers with percentages. These analyses were conducted using IBM SPSS Statistics version 25.

## Results

### Participant and interview characteristics

Fourteen participants were interviewed. One interview was excluded from the analyses because the participant (who had a low risk result from geriatric screening) did not understand the procedure of the interview, and did not answer any of the questions. In total, 13 participants were included, with a median age of 82 years (range 71–94), of whom 7 (54%) were female (Table 1). Twelve interviews took place at the participants’ homes, and one interview took place in a geriatric rehabilitation center. In 8 interviews (62%), a family member was present and participated during the interview. These family members all had been present during the ED visit as well. The participants had a broad range of chief complaints at ED arrival and 7 participants (54%) required very urgent care. In total, 6 participants (46%) had a high risk screening result, of whom 2 participants on the functional domain and 4 participants on the cognitive domain.

**Table 1:** Participant and interview characteristics

	Demographics		Characteristics of the ED visit				Characteristics of the interview	
	Sex	Age	Chief complaint at ED arrival	Triage urgency^a^	Result of APOP screening	Hospital admis- sion	Days between ED visit and interview	Family member present during interview
1	Male	78	Dyspnea	Orange	Low risk	No	22	Yes
2	Female	71	Fall, wrist fracture	Yellow	Low risk	Yes	28	No
3	Male	78	Chest pain	Yellow	Low risk	No	32	Yes
4	Female	82	Malaise	Orange	Low risk^b^	Yes	34	No
5	Female	71	Malaise	Yellow	Low risk	No	38	Yes
6	Male	76	Chest pain	Orange	High risk on cognitive domain	No	36	Yes
7	Female	87	Chest pain	Yellow	Low risk	No	42	No
8	Male	84	Dyspnea	Orange	High risk on functional domain	Yes	44	No
9	Female	90	Collapse	Yellow	High risk on functional domain	No	51^c^	No
10	Female	75	Head/brain injury	Orange	High risk on cognitive domain	No	42	Yes
11	Female	82	Head/brain injury	Green	High risk on cognitive domain^d^	No	42	Yes
12	Male	94	Suspected dissection aorta	Orange	High risk on cognitive domain	No	42	Yes
13	Male	94	Hip luxation	Orange	Low risk	No	42	Yes

^a^ Triage urgency according to the Manchester Triage System: Green = standard care > 1 h, Yellow = urgent care < 1 h, Orange = very urgent care < 10 min

^b^ Screening was incorrectly completed, the participant turned out to have a high risk on the functional domain

^c^ Interview took place in a geriatric rehabilitation center

^d^ Participant with diagnosis of dementia

#### Theme 1. Experiences with geriatric screening in the ED

##### Recall of screening administration

The majority of participants had no direct recall of the administration of geriatric screening questions in the ED during the triage process. They did not experience screening as a separate part of ED care. Some participants without recall of the screening, could also not remember other (large) parts of their ED visit due to pain, shortness of breath or other complaints. The overwhelming impressions they had during their ED visit resulted in difficulties remembering what had happened at the time.

*“Actually not much, because I wasn’t really approachable [ … ] They asked me a couple of questions, but those got a bit lost as I was so out of breath that I wasn’t really registering much. So, I don't actually know much about that anymore.”* [P8]

Some participants were able to share their experiences with the screening administration without further explanation. One participant had recognized geriatric screening as something new because the questions had not been asked during his previous ED visits. Most participants without direct recall did remember the screening after we showed them the video explaining the APOP screening program. The questions testing cognition were remembered mostly, especially the question to list the months in reversed order. None of the participants objected to answering cognition questions, although one participant indicated that it was difficult to answer these kind of questions in a hectic ED environment. Participants often explained that they were glad that they answered the cognition questions right.

*“Now I remember what they asked. She asked me to list the months in reverse order from December. [ … ] Thankfully my brain is still working fine.”* [P2]

According to the participants, the screening results were not shared with them. It remained unclear whether the results were indeed not shared or that the participants could not remember them being shared. The participants stated that they did not miss this, because they believed that the results were good and there was nothing to discuss. However, some of these participants had a high risk screening result. Some participants stated that the screening results are only important for care providers, but not for patients themselves.

“*No, that's your job, right? The doctor is supposed to know what is going on. If I have to be the one thinking of that, well, then I wouldn't have much of a life left over.”* [P6]

##### Experienced consequences of screening

None of the participants experienced negative consequences from screening. Some participants reported that they had experienced a positive consequence of screening on the care they received. They were positive about the screening questions being asked because they thought it provided care providers a complete picture of them as a patient. Among this group, the division of high- and low-risk screened participants was equal.

*“It didn't bother me. At least then they have the full picture of me as a patient. [...] They asked me some specific things, like how am I doing, how do I feel. Well, that does calm you down.”* [P1]

Another positive consequence experienced was a perceived feeling of safety at discharge, since the home situation was checked thoroughly. Additionally, participants with a high risk screening result who were discharged home, were satisfied with the telephone call they received after discharge.

“*The other day someone from the hospital called. That was something new for me. ‘How are you Mister [ … ]?’ I thought that was amazing!”* [P12]

#### Theme 2. Attitude towards geriatric screening of older ED patients

##### General attitude towards screening

The overall attitude of participants towards screening older patients for high risks on adverse outcomes or ‘frailty’ in the ED was positive. None of the participants had a negative attitude towards screening. One participant described screening as understandable.

*“Well I think it’s only positive. You will find out more about a person by knowing the background.”* [P5]

It was mentioned that although the intentions of geriatric screening are good, care providers should be aware that they use the results rightfully. A ‘frail’ result from screening should not result in communicating with older patients in a childlike manner or treating them as if they are piteous.

*“Sometimes they call you mummy and that sort of things. I know they mean well, but I just want … I’m still fully here* [points at head] *and I just want to be treated as a normal person.”* [P9]

Additionally, care providers must ensure that older patients can participate in decision making independent of their screening result. None of the participants thought that geriatric screening is discrimination on age. However, it was mentioned by some participants that screening might be beneficial for all patients, regardless of age.

*“But I think, purely speaking about the ED, it would be good to do the same screening for everyone who is approachable, let’s say from the age of 12 or 14.”* [P1]

Some participants described the importance of asking older people specifically about their frailty. Otherwise, people might not mention frailty themselves due to the fact that they feel ashamed or they don’t think it is important. Specifically asking all older patients about their frailty was mentioned to be important because it is difficult for care providers to estimate for whom it is necessary.

*“Examine the whole situation, and do that for everybody [ … ] It is better to ask one question too many, than one question too few. You can’t see from the outside whether or not it is needed [ … ] And a lot of people would never mention it themselves, especially because it wasn’t a topic that could be discussed in earlier times. People have learned to just keep quiet and don’t … well, complain.”* [P2]

##### Added value of screening

Although most participants had not experienced consequences from screening themselves, they could describe the possible added value for other older ED patients. First, screening could help care providers to assess older patients holistically. Participants encouraged a holistic approach of older patients with attention for the social background, besides the medical problems.

*“I think it is very important that they have a complete view of the background of older people [ … ] The doctor is focused more on the medical part, but a person’s background, someone’s lifestyle, that sort of things, I think that is also very important information.”* [P8]

Second, the early recognition of geriatric problems was mentioned as an added value of screening. Recognizing cognition problems was found to be important for ED care providers to indicate whether someone understands the information being given. Additionally, information about frailty was found to be as important as medical information and it should be shared with other care providers.

*“Someone might be having some cognitive problems without being aware of it. This could be a very early recognition [ … ] Care providers could pass on this information to the GP, so that there can be a follow-up.”* [P2]

Third, geriatric screening could be used to comfort patients by means of good communication and attention. The attitude of care providers in communicating with older patients was also stated as one of the main factors for satisfaction with received care in the ED.

*“Attention, that is the most important for people. That someone immediately addresses you correctly. [ … ] That you are made at ease.”* [P1]

##### Defining frailty

Although the general attitude towards screening for ‘frailty’ in the ED was positive, participants found it difficult to define what it is we should screen for. Frailty was a hard to define concept for which all participants gave different definitions (see Table 2). Participants who found it hard to define frailty and participants who did not consider themselves to be frail, had difficulties explaining the added value of geriatric screening as well.

**Table 2:** Overview of the definition and perception of frailty for all participants

	Frailty definition	Perception own frailty in general
1	Immobility, diseases, dependence, in need for care	*“Not at all! Absolutely not! [...] No, I am still fit. I still do all kinds of things for other people and for myself. No, I am not frail.”*
2	Poor mental functioning, loneliness, dependence, not being able to stand up for yourself	*“I feel frail on the street right now … I have to overcome my fear of falling.”*
3	Forgetting things, communication problems, not being yourself, is a part of ageing	*“I feel frail because I am often searching for things and I can’t figure it out by myself […*] *I am not very mobile anymore and I regularly need oxygen, that is frailty. But, well, that’s part of [getting older]”*
4	Poor physical functioning, dementia, dependence on transport, being piteous	*“No! No, absolutely not. They shouldn’t be feeling sorry for me. No, really not! That really makes me angry.”*
5	Loneliness, falls	*“I feel frail when I want to go for a walk but I can’t. I need someone who says, ‘Hey, I will pick you up’ […] At that point you are actually frail, because you are by yourself.”*
6	Poor physical functioning, dependence on transport	*“Frailty, I would find that terrible. I don’t think I’d just admit to that […] I don’t feel frail.”* Partner: *“He still drives a car, he still drives a scooter. We still do everything ourselves.”*
7	In need for care	*“I take showers by myself, I can still take care of myself completely, I don’t struggle with anything […] I am not frail.”*
8	Not being able to do groceries, falls	*“No, the GP also says, you are very flexible for your age […] I do have some health issues which is not good. That’s what’s holding me back. […] But no, I do not feel frail.”*
9	Immobility, poor mental functioning, lack of resilience	*“I still follow everything, the news. And I solve lots of puzzles. But I can’t go out and that’s difficult, you could call that a sort of frailty. […] When everything is decided by others, then I feel a bit left out […] Yes, you are more frail than a healthy, young person, but that only makes sense.”*
10	(could not give a definition)	*“No, not me, no […] I do not feel frail, no […] Yes, some days you do, of course.”*
11	Immobility, communication problems due to dementia, lack of resilience	*“Occasionally, yes. Because then I think, ‘Oh no, are they going to ask me that?’”* Partner: “*She can’t always participate in entire conversations, and if she wants to tell something, she can’t anymore. Then she loses the plot a bit […] She has a bit, a little bit, of dementia … and then she can’t remember sometimes.. a little Alzheimer’s.”*
12	Falls, diseases, dementia, loneliness and not being yourself, is a part of ageing	*“We are frail, of course. Our friends who all have died by now, that may also happen to us […] We think about our frailty, but I still drive my car and frailty is more important to my sons because they believe I shouldn’t drive anymore.”*
13	Immobility, poor mental functioning, lack of resilience, in need for care	Partner: *“Yes, physically of course. He is not walking well, and he is frail when walking on the street”* Participant: *“Well, yes and no. I am naturally stubborn […] and I have often had the feeling, in retrospect, I shouldn’t have done that, you are taking a risk.”*

*“To be honest, I doubt whether you can do anything with it. What should you do with it? Examining frailty. Do we even know what frailty is? And what are you going to do about it? [ … ] I don’t know much about it yet, but I will know it when I am at that point myself.”* [P4]

The perception of participants’ own frailty depended on their given definition. Some participants did not meet their own definition and therefore did not feel frail. Others generally did not consider themselves frail, but contradictory did mention their own situation as examples of frailty to provide a definition. The majority of participants felt frail to a greater or lesser degree. Some participants felt frail in general, others gave examples of frailty in particular situations. For example, a visit to the ED was mentioned as a moment when you can feel frail. One participant explained that the presence of a caregiver could reduce this feeling of frailty in the ED. The most commonly mentioned definitions of frailty were: poor physical functioning or immobility, poor mental functioning or dementia, dependence or in need of care, loneliness, and lack of resilience. Some participants stated that frailty is a normal part of ageing, while others did not want to have the label ‘frail’ because they feel ashamed or don’t want to be found piteous.

## Discussion

This qualitative study is the first to explore perspectives of older patients on the use of geriatric screening in acute care. This study shows that older patients had predominantly positive experiences and attitudes towards the use of geriatric screening in routine ED care. Geriatric screening was considered as a normal part of routine ED care and most participants believed that screening contributes to assessing older patients holistically, recognizing geriatric problems early and comforting patients with communication and attention.

The experiences of the participants with geriatric screening during their ED visits were good, and none of the participants experienced screening as negative, unpleasant or burdensome. Literature suggests that older people tend to be positive about their received ED care [22–24], and these findings are in line with our results. The participants’ positive experience with their ED visit may have influenced their experience with screening, because screening was not perceived as separate part of ED care. Furthermore, none of the participants objected to answering questions testing cognition, which is an interesting finding because we know from previous research that care providers experienced barriers asking these questions to older patients [16]. This finding underlines the importance of investigating and incorporating not only the care provider perspective, but also the often underexposed patient perspective.

In this study, we explored both experiences with geriatric screening and the overall attitude towards screening in routine ED care. It is possible that the attitude towards screening was influenced by the positive experiences with geriatric screening in the ED, but most likely also by the perception of participants’ own frailty. Participants who did not feel frail themselves found it more difficult to describe the added value of screening for patients and care providers. It is unlikely that the attitude towards screening was influenced by the screening results itself, because the results were unknown to the patients. Moreover, both high risk (‘frail’) and lowrisk (‘non-frail’) screened participants had a positive attitude towards screening and described the potential added value of screening. Furthermore, participants described the importance of a holistic approach to unravel the older patient as a whole beyond just the medical complaints. The need of older people to receive holistic care and to be involved in decision-making has been described previously for the ED setting [25], and is corresponding to literature in community-dwelling older people and older patients in regular health care [26–29]. Our findings suggest that screening could aid in reaching this goal and additionally could help to comfort patients by means of attention for them as a person.

Frailty was a hard to define concept for the participants, which is in line with previous studies showing that both older people and care providers find it hard to define frailty [4, 30, 31]. Literature suggests that there is a difference in objectified frailty - ‘being frail’ - and how older people perceive their own frailty - ‘feeling frail’ [32]. Although this difference was not explicitly discussed in the interviews, this suggestion is in line with our results that showed that in more than half of the participants the result from screening did not match the perceived frailty of the participant. Furthermore, participants stated that care providers should use the screening results rightfully in their communication with frail patients. In line with literature showing that the label ‘frail’ could be experienced negatively [8, 29, 33], we found that some participants explicitly did not want to be labelled frail, for example because they did not want to be found piteous. However, none of the participants thought that geriatric screening is discrimination on age and they even believed that screening might be beneficial for all patients, regardless of age. More importantly, despite the sometimes difficult and negatively experienced concept of frailty, all participants were positive about continuing the use of geriatric screening in routine ED care. So although the term ‘frailty’ was often not something that participants wish to associate themselves with, because of the stereotypical images that the concept evokes (Table 2), the concept of identifying patients by measuring frailty to tailor care to the individual patients was well accepted.

This qualitative study adds valuable new information for clinical ED practice about the patient perspective on the use of geriatric screening and advocates for continuing the implementation of screening in routine practice. The results of this study might also influence the public debate in favor of using screening. Older patients had predominantly positive attitudes towards the use of geriatric screening in the ED. We will therefore continue the use of the APOP screening program in our hospital and recommend other hospitals to implement geriatric screening in the ED as well. Our study shows that sharing the screening results with patients in the ED may not be necessary as long as the results are handled properly and care providers respectfully communicate with frail older patients and involve them in decision-making. Small actions such as arranging the presence of an informal caregiver, may already make the patient feel less frail in the ED, and could therefore be incorporated in screening programs. Future research might be needed to evaluate the experiences of older patients with other screening instruments.

Strengths of this study can be accounted to the novelty of exploring the patient perspective of geriatric screening in the ED. In addition, the older patients’ experiences were not evaluated in a research setting, but during routine ED care visits. Finally, a heterogeneous group of participants was included in this study by using purposive sampling. This study also has several limitations. First, since the results relate to a small number of older ED patients from one academic hospital in the Netherlands, these are not generalizable to a global ED population. However, the number of participants was adequate for the purpose of this qualitative study and saturation of data was reached within a heterogeneous group of participants. Besides, the insights and experiences are likely to have transferable similarities for other older ED patients. Second, some of the participants could not remember screening being executed in the ED, which might be caused by good implementation or by recall bias. The interviews were planned as soon as possible, but due to sufficient recovery time and logistic reasons there was an average period of 5 weeks between the ED visit and the interview. Third, family members actively participated in the interviews which could have influenced the described experiences and attitudes of the patients. However, almost all family members were older people themselves and they all had been present during the ED visit, which made their opinion of added value as well. Fourth, the APOP screening instrument was used to explore the patients’ perspective about geriatric screening, while this is technically not a frailty screener but a risk stratification instrument which identifies older patients at high risk of adverse outcomes. A high risk screening result was used as a proxy for ‘frailty, which means that other screening instruments might have selected a slightly different group of people as being ‘frail’. We used the APOP screener because this instrument was implemented in routine ED care in our hospital, and we aimed to explore patients’ experiences with geriatric screening in a real-life setting.

## Conclusions

From an ED-patients’ perspective, geriatric screening was experienced as a normal part of ED care and was considered to be of added value. Older patients stated that screening contributes to assessing older patients holistically, recognizing geriatric problems early and comforting patients with communication and attention. The results from this qualitative study advocate for continuing the implementation of screening in routine ED practice.

## Supplementary Information

**Additional file 1.** Additional information about the APOP screening program.

**Additional file 2.** Interview topic list.

**Additional file 3.** Consolidated Criteria for Reporting Qualitative Studies (COREQ) checklist.

## Data Availability

The datasets used and analysed during the current study are available from the corresponding author on reasonable request.

## Abbreviations

APOP: Acutely Presenting Older Patient
ED: Emergency Department
GP: General Practitioner
LUMC: Leiden University Medical Center
