# Supplementary material for: Experiences with and attitudes towards geriatric screening among older emergency department patients: a qualitative study
Source: BMC Geriatr. 2021 Mar 20;21:198. doi: 10.1186/s12877-021-02144-7 (PMC7981953; doi:10.1186/s12877-021-02144-7)
Supplement: Supplementary file 1 — Additional file 1. Additional information about the APOP screening program. [file 12877_2021_2144_MOESM1_ESM.docx]

**ADDITIONAL FILE 1 –** Additional information about the APOP screening program

The APOP screening program is developed for ED patients aged ≥70 years and consists of three parts (visualised in the figure below):


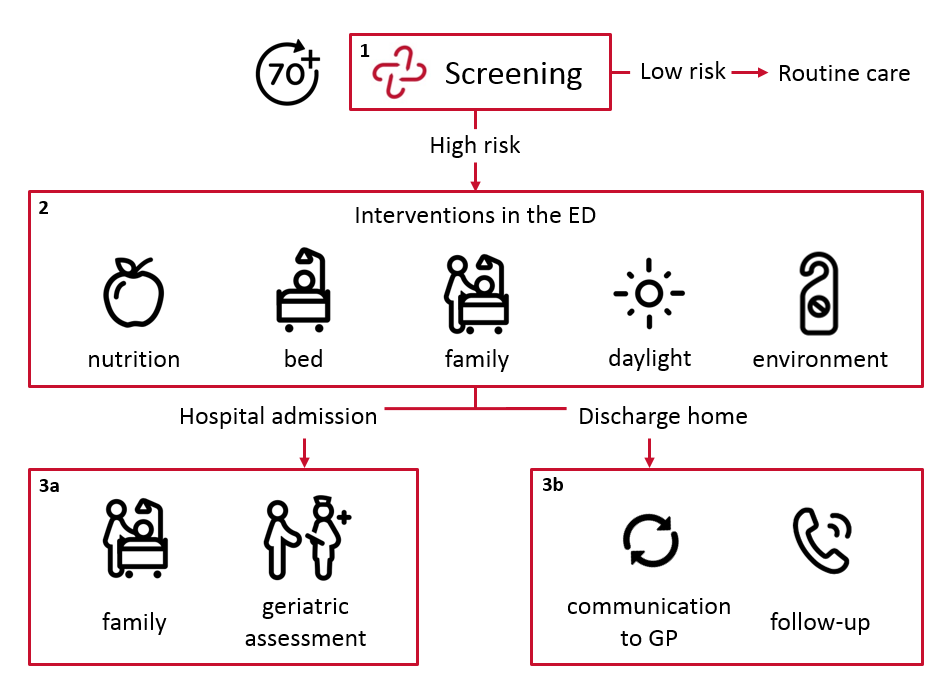


1. Screening

The APOP-screener can be administered in 90 seconds and identifies the patients’ individual risk of 90-day functional decline and/or mortality and signs of impaired cognition in the ED.[18] All patients aged ≥70 years are eligible for screening after routine ED triage. Only patients with unstable medical conditions (Manchester Triage System category “red”: i.e. major trauma, resuscitation, thrombolysis) are not screened directly at triage, but can be screened later during their ED visit. The APOP screener and the screening results are incorporated in the hospital’s electronic health records (EHRs) and are visible for all care providers.[16,17] Patients with a low risk according to screening receive routine care. Patients are considered ‘ frail’ or ‘high risk’ when having a 45% or higher risk of functional decline and/or mortality within 90 days or when having signs of impaired cognition. This applies to roughly 30% of the older ED population. The nine questions of the APOP screener are visualised in the following table.

| **The Acutely Presenting Older Patient (APOP) screener** | |
| --- | --- |
| **Questions** | **Predictors** |
| Filled out by the triage nurse: |  |
| 1. What is the age of the patient? | Age (per 5 years increase) |
| 1. What is the gender of the patient? | Male |
| 1. Did the patient arrive by ambulance? | Arrival by ambulance |
| Asked to the patient: |  |
| 1. Before the illness or injury that brought you to the ED, did you need someone to help you on a regular basis? (like housekeeping, preparing meals) | Need help prior to ED visit (IADL) |
| 1. Before the illness or injury that brought you to the ED, did you need assistance in bathing or showering? | Need help bathing or showering |
| 1. Have you been hospitalized during the past six months? | Hospitalized past six months |
| 1. Are you diagnosed with dementia? | Impaired cognition* |
| 1. What year is it now? |  |
| 1. Say the months in reversed order |  |
| Abbreviations: ED = emergency department, IADL = instrumental activities of daily living.  *: Cognition is considered to be impaired when the patient is diagnosed with dementia (question seven) or when the patient incorrectly answers question eight or nine.  Prediction model: 1/(1+exp(-(-5.848 + 0.262 x ‘(age/5)’ + -0.072 x ‘male’ + 0.460 x ‘arrival by ambulance’ + 0.534 x ‘need help prior to ED visit’ + 0.567 x ‘need help bathing or showering’ + 0.432 x ‘hospitalized past six months’ + 0.255 x ‘impaired cognition’)))  Application: ‘http://screener.apop.eu/’ | |

2. Interventions for high risk screened patients in the ED

A high risk result from screening leads to follow-up actions and interventions. Physicians and nurses are advised to execute interventions in the ED to increase comfort, family involvement and delirium prevention. The interventions of the program were based on recommendations from international geriatric emergency medicine guidelines and were adjusted for use in the Dutch ED setting.[1]

3a. Interventions for high risk screened patients admitted to the hospital

Interventions can be conducted in an early phase after high risk patients are hospitalised. Care providers are advised to avoid a prolonged ED length of stay and to arrange family involvement during transfer to the ward. The geriatric consulting team is informed automatically by the EHRs to arrange a comprehensive geriatric assessment during hospital admission.

3b. Interventions for high risk screened patients discharged home from the ED

High risk screened patients who are discharged home from the ED receive a telephone call within 24 hours after discharge by one of the ED nurses to inform about remaining questions about their ED treatment and the need for additional support (i.e. clarification of instructions). The general practitioner (GP) is informed about the screening result automatically by the EHRs in the discharge letter from ED physicians.
