# Supplementary material for: Experiences with and attitudes towards geriatric screening among older emergency department patients: a qualitative study
Source: BMC Geriatr. 2021 Mar 20;21:198. doi: 10.1186/s12877-021-02144-7 (PMC7981953; doi:10.1186/s12877-021-02144-7)
Supplement: Supplementary file 2 — Additional file 2. Interview topic list. [file 12877_2021_2144_MOESM2_ESM.docx]

**ADDITIONAL FILE 2 –** Interview topic list

**Topic 1. Experiences of the Emergency Department (ED) visit**

Example questions:

- Why did you visit the ED?
- What do you remember about your ED visit?
- How did you experience this ED visit?
- Did you understand everything that happened and what was being said to you?
- How were your wishes, expectations and personal situation taken into account? [topic 4]
- In retrospect, what could have been done differently?
- What was your experience with being discharged? What made you feel safe to go home?

**Topic 2. Experiences with geriatric screening in the Emergency Department**

Example questions:

- What have you noticed about questions being asked to screen for frailty?
- How did you experience being asked these questions?
- What was communicated to you about the screening results?
- What are your feelings towards how healthcare professionals acted upon the screening results?
- In your experience, what was the added value of the screening?

*Video being shown which explains the APOP screening program*

**Topic 3. Attitude towards geriatric screening in the Emergency Department**

Example questions:

- Which situations do you recognize from this video? [topic 2]
- Why do you think the ED screens for frailty?
- What is your definition of frailty?
- To what extent do you feel frail yourself? (in general, in the ED, in certain situations)
- What are your feelings towards frailty screening in older patients at arrival in the Emergency Department? [topic 4]
- How could frailty screening be of importance?
- How do you think this screening can help to improve care for the older patient?
- What can be the added value of using frailty screening?

**Topic 4. Needs and goals of older patients in the Emergency Department**

*Additional topic – only use if there is enough time*

Example questions:

- What is important for older patients who visit the Emergency Department? What are their needs?
- How are the needs of older patients different from younger patients?
- Why is it important to take differences in needs between patients into account?
- Which goals should we pursue for older patients in the Emergency Department?
- What should be the goal(s) for older patients during their treatment in the Emergency Department? And after their visit? Different from younger patients?
- What was your experience: how did your wishes and personal situation were taken into account? [topic 1]
